# Development of Quercetin Solid Dispersion-Loaded Dissolving Microneedles and In Vitro Investigation of Their Anti-Melanoma Activities

**DOI:** 10.3390/pharmaceutics16101276

**Published:** 2024-09-30

**Authors:** Monsicha Khuanekkaphan, Kesinee Netsomboon, Adryan Fristiohady, Rathapon Asasutjarit

**Affiliations:** 1 Thammasat University Research Unit in Drug, Health Product Development and Application (DHP-DA), Department of Pharmaceutical Sciences, Faculty of Pharmacy, Thammasat University, Pathum Thani 12120, Thailand; monsicha.khu@dome.tu.ac.th; 2 Department of Pharmaceutical Sciences, Faculty of Pharmacy, Thammasat University, Pathum Thani 12120, Thailand; nkesinee@tu.ac.th; 3 Department of Pharmacology and Clinical Pharmacy, Faculty of Pharmacy, Universitas Halu Oleo, Kendari 93132, Indonesia; adryanfristiohady@uho.ac.id

**Keywords:** quercetin, microneedles, solid dispersion, melanoma, transdermal drug delivery

## Abstract

**Background:** Melanoma is a skin cancer that requires early treatment to prevent metastasis. In particular, the superficial spreading melanoma, excisional surgery with local administration of anti-cancer drugs via microneedles is currently considered a potential combination therapy. Quercetin is a natural flavonoid having activities against melanoma cells. Unfortunately, the therapeutic effect is limited by its poor water solubility. **Objectives:** This study aimed to develop formulations of solid dispersion-loaded dissolving microneedles (SD-DMNs) of quercetin and to investigate their in vitro activities against melanoma cells. **Methods**: Quercetin solid dispersions (Q-SDs) were prepared using polyvinylpyrrolidone K30 (PVP) via a solvent technique. The optimized Q-SD was selected for preparing Q-SD-loaded dissolving microneedles (Q-SD-DMNs) using a mold casting method. **Results**: Q-SDs had higher water solubility than that of quercetin by 5–10 times depending on the ratio of quercetin-to-PVP. The presence of quercetin in the Q-SD and Q-SD-DMN were in an amorphous form. The obtained Q-SD-DMNs had pyramid-shaped microneedles. Their strength depended on the compositions, i.e., ratios of hyaluronic acid-to-sodium carboxymethylcellulose and the content of Q-SD. An optimized Q-SD-DMN increased the in vitro skin permeation of quercetin compared to that of microneedles containing quercetin (without being processed). From the molecular investigations, the optimized Q-SD-DMN reduced the viability of the A375 cells (melanoma cells) through the induction of cell apoptosis. It suppressed Bcl-2 gene expression and led to a lower content of Bcl-2 in the cells. **Conclusions:** The optimized Q-SD-DMN has a potential for use in further in vivo studies as a synergistic method of melanoma treatment.

## 1. Introduction

Skin cancer is a common cancer found globally. It is categorized into two types: melanoma and non-melanoma. Melanoma is an aggressive skin cancer characterized by the uncontrolled proliferation of melanocytes. In contrast, non-melanoma skin cancers, such as basal cell carcinoma and squamous cell carcinoma, tend to grow slowly and are highly treatable [1]. Nowadays, the incidence of melanoma has been rising, with estimates of around 510,000 new cases throughout the world by 2040 [2]. Some contributing factors have been identified, e.g., UV radiation exposure and family history [3].

Treatments for melanoma vary in accordance with the stage of the disease [4]. They include surgery, chemotherapy, radiotherapy, gene therapy, immunotherapy, and a combination of these procedures [5]. For superficial spreading melanoma (SSM), which is the most common subtype of melanoma [3] with vertically invasive growth from a radial growth stage, excisional surgery is considered the first choice of treatment at the early stages (stage I and II). This procedure is suitable for tumors with 1–4 mm thickness. It requires accurate diagnosis and precise tumor size measurement to ensure complete tumor tissue removal [3,5]. Currently, some additional treatments have been used to promote the cure rate of SSM, in particular, the application of microneedles containing anti-cancer drugs. They have shown effectiveness for local treatment of SSM as a locally targeted therapy. They have also been considered as a synergistic therapy for some patients who cannot receive only surgical excision for treatment of SSM [5,6,7]. 

Many publications have indicated that microneedles can effectively penetrate through the epidermis and deliver anti-cancer drugs, for example, paclitaxel, doxorubicin, and cisplatin, to the targeted skin layers [8]. They also reduce the risk of bleeding, pain, and systemic side effects from such anti-cancer drugs [5,8]. In particular, dissolving microneedles that contain drugs dispersed in a water-soluble matrix have been recognized as a potential drug delivery system for transdermal local treatment because they effectively increase drug concentration at the targeted skin tissues. Moreover, they possess some advantages over other kinds of microneedles because of their biocompatibility, safety, and ability to control drug release. Furthermore, they can be prepared easily using a simple mold casting method [9,10,11]. Unfortunately, dissolving microneedles are suitable for hydrophilic drugs because of the hydrophilic properties of the water-miscible matrix of the microneedle bases [10]. Thus, they are limited to highly lipophilic drug delivery. Therefore, the water solubility of lipophilic drugs should be optimized not only to increase the effectiveness of their percutaneous absorption but also to obtain homogeneity of the products when the particular drugs are loaded into the base matrix of dissolving microneedles [12]. 

One interesting process to improve the water solubility of lipophilic drugs is solid dispersion using a solvent method, in which the drug solution in a suitable vehicle is dispersed into water-soluble carriers [13]. Due to its convenient production process without the use of toxic organic solvents and complicated chemical reactions, solid dispersion has been widely used in the pharmaceutical industry [14]. 

Quercetin is a natural flavonoid that is found in various vegetables and fruits, including some medicinal plants, e.g., *Hypericum perforatum* (St. John’s wort) and *Sambucus canadensis* (elderberry). It exhibits promising anti-inflammatory, antioxidant, and anti-cancer activities [15]. For anti-melanoma activities in particular, quercetin has inhibitory activities on oxidative stress enzymes that lead to modulating DNA methylation in the melanocytes [16]. It can induce apoptosis in melanoma cells by the downregulation of B cell lymphoma 2 (Bcl-2), which is a crucial anti-apoptotic protein that regulates cell death and plays a pivotal role in cancer progression [17]. Therefore, quercetin has the potential to be used as a supportive anti-melanoma agent to reduce the use of some anti-cancer drugs that possess serious systemic adverse reactions [16]. However, quercetin has poor water solubility, which causes poor oral bioavailability [18]. An appropriate drug solubility improvement technique, such as solid dispersion, should be applied to overcome this problem. Furthermore, local administration of quercetin using microneedles for transdermal delivery of quercetin directly to the SSM lesion is one possible strategy to increase the local concentration of quercetin in a specific skin area. 

To date, there is still a lack of data on quercetin-loaded dissolving microneedles (Q-DMNs) preparation for local treatment of melanoma. This study, thus, aims to (1) improve the water solubility of quercetin by using the solid dispersion process, (2) prepare quercetin solid dispersion-loaded dissolving microneedles (Q-SD-DMNs), and (3) investigate in vitro inhibitory activities of Q-SD-DMNs on the melanoma cells.

## 2. Materials and Methods

### 2.1. Materials

Quercetin (≥95% purity), polyvinylpyrrolidone K30 (PVP) with an average molecular weight of 40,000, sodium carboxymethylcellulose (Na-CMC), and 3-[4,5-dimethylthiazol-2-yl]-2,5-diphenyltetrazolium bromide (MTT) were supplied by Sigma-Aldrich (St. Louis, MO, USA). Diethylene glycol monoethyl ether (DEGEE) (≥99% purity), hyaluronic acid (HA), and solvents for a high-performance liquid chromatography (HPLC) analysis, i.e., methanol and orthophosphoric acid were purchased from TCI (Tokyo, Japan) and Merck KGaA (Hessen, Germany), respectively. They were all analytical grade and used immediately after being received. 

### 2.2. Cell Cultures

Human skin fibroblast cells (HFF-1 cells; SCRC-1041) and human malignant melanoma cells (A375 cells; CRL-1619) were purchased from the American Type Culture Collection (ATCC, Manassas, VA, USA). Dulbecco’s modified Eagle’s medium (DMEM), penicillin-streptomycin, trypsin-EDTA, fetal bovine serum (FBS), and phosphate-buffered saline (PBS) for cell cultures were acquired from Gibco (Big Cabin, OK, USA). 

The A375 cells were cultured in DMEM supplemented with 1% penicillin-streptomycin and 10% FBS, while the HHF-1 cells were cultured in DMEM supplemented with 1% penicillin-streptomycin and 15% FBS. All cells were incubated at 37 °C under a 5% CO_2_ atmosphere.

### 2.3. Preparation of Quercetin Solid Dispersion

In this study, a solid dispersion process was applied to improve the solubility of quercetin before incorporating it into the dissolving microneedle base matrix. Solid dispersions of quercetin were prepared by following the solvent method that was previously reported with some modifications [19]. Briefly, quercetin was dissolved in DEGEE (0.1 g) and slowly mixed with PVP at various ratios of quercetin-to-PVP, as shown in Table 1. After that, purified water (4.0 g) was added and mixed. The mixtures were then kept at −80 °C for 24 h before being subjected to freeze-drying for 48 h (−80 °C, 0.002 mbar) using a freeze dryer (Martin Christ, Osterode am Harz, Germany). The obtained freeze-dried quercetin solid dispersions (Q-SDs) were stored in a vacuum desiccator (Kartell, Milan, Italy) to ensure complete dehydration before use in further experiments. The yield percentage of obtained Q-SDs was calculated using Equation (1):

(1) $$\%\;\mathrm{Yield}=\frac{\mathrm{total weight of obtained freeze-dried products}}{\mathrm{total weight of Q-SD compositions before freeze-drying}}\times \;100$$

In addition, a physical mixture containing the same ingredients as the optimized solid dispersion was prepared as a representative. It was called quercetin physical mixture (Q-PM) and prepared by simply mixing quercetin with PVP and DEGEE using a mortar and pestle. 

### 2.4. Content Uniformity

This experiment was conducted to determine the content of quercetin in Q-SDs and Q-PM after the preparation. A high-performance liquid chromatography (HPLC) analysis was applied using the following conditions. 

An accurate amount of the samples (5 mg) was completely dissolved in 5 mL of a mobile phase containing methanol and 0.1% orthophosphoric acid at a ratio of 65:35. Then, it was filtered through a 0.45 μm membrane and injected into an HPLC instrument (Shimadzu-SPD-20A, Shimadzu, Japan). Chromatographic separation was achieved using an Inertsil TM C18 column (4.6 × 250 mm, 5 µm). Detection was performed with a UV-Vis detector (Hitachi U-2000, Tokyo, Japan) set at a wavelength of 369 nm. The acceptable range of content uniformity was between 90 and 110% of the theoretical amount of quercetin in the samples.

### 2.5. Determination of Water Solubility

In order to compare the water solubility of quercetin and that of the quercetin in the samples, a solubility test was performed following the protocol that was previously described [20]. Briefly, 5 mg of quercetin (a raw material) and the samples, i.e., Q-SDs and Q-PM that contained the same content of quercetin, were dispersed in purified water (5 mL). Test tubes containing these particular dispersions were shaken at the average room temperature in Thailand (27 ± 1 °C) at 100 rpm for 24 h. Then, they were centrifuged at 25 °C, 15,000 rpm for 10 min. The supernatant was filtered through a 0.45 µm membrane and analyzed for quercetin content using the HPLC conditions shown in Section 2.4.

### 2.6. Characterization of Quercetin Solid Dispersions

#### 2.6.1. PXRD Analysis

The PXRD analysis was conducted to evaluate the crystallinity of quercetin in the samples using an X-ray diffractometer (Rigaku-Miniflex 600 Benchtop, Rigaku Corporation, Tokyo, Japan) with copper-Kα radiation at a voltage of 30 kV, 15 mA. The samples were tested at 2θ angles ranging from 5° to 40°, with a scan step size of 0.02° and a scanning speed of 5°/min [21].

#### 2.6.2. DSC Analysis

The DSC analysis was performed to investigate the thermal properties of the samples via a differential scanning calorimeter (DSC 3+ STARe System, Mettler Toledo, Baden-Wurttemberg, Germany). For this analysis, 5 mg of each sample was placed in an aluminum pan and sealed with a perforated cover, while an empty aluminum pan served as a reference. Nitrogen gas was purged at a flow rate of 20 mL/min. Subsequently, the samples were heated from 25 °C to 300 °C at a heating rate of 10 °C/min to observe thermal transitions [21].

#### 2.6.3. FTIR Analysis

The FTIR spectroscopy technique was employed to investigate functional groups of quercetin and the ingredients consisting of the samples by using an FTIR spectrometer equipped with an attenuated total reflection (ATR) accessory (Dia/ZnSe) (IRTracer-100, Shimadzu, Tokyo, Japan). The IR spectra were recorded in the wavenumber range of 600–4000 cm^−1^, with a total of 30 scans and a spectral resolution of 4 cm^−1^ [21].

### 2.7. Morphology Observation

The morphology of the samples was observed under a scanning electron microscope (SEM) (JSM-5410LV, JEOL, Tokyo, Japan). The samples were coated with a thin layer of gold to enhance conductivity and obtain clear images. The analysis was performed with a beam voltage of 15 kV, 12 mA [22].

### 2.8. Development of Quercetin Solid Dispersion-Loaded Dissolving Microneedles

Formulations of Q-SD-DMNs were developed based on the selected base formulation of dissolving microneedles (B-DMNs) from the optimization process. 

B-DMNs were prepared by dissolving HA and Na-CMC in purified water (20 mL) at various ratios of HA-to-Na-CMC, i.e., 2:1, 1:1, and 1:2, respectively. Then, the solutions were poured into polydimethylsiloxane molds that contained 15 × 15 arrays of pyramid-shaped needles. Each needle had a base dimension of 200 × 200 μm and a vertical height and slant height of 600 and 500 μm, respectively. Then, the molds containing B-DMN solutions were subjected to pressure of 1 bar for 2 min and dried in a desiccator for 24 h. The morphology of the obtained B-DMNs was observed using a light microscope (Dino-Lite Premier AM4113ZT, AnMo Electronics Corporation, Taipei, Taiwan) [9]. An optimized B-DMN formulation was selected from the penetration efficiency. 

Q-SD-DMNs were prepared by adding the optimized Q-SD into a solution of the selected B-DMNs at weight ratios of Q-SD-to-B-DMN of 0.5:99, 1:99, and 1.5:99, respectively. 

In this study, Q-DMNs were also prepared by loading a suspension of quercetin (without being processed) that contained the same equivalent weight of quercetin as the optimized Q-SD-DMNs into the selected B-DMN solution using the same mold.

### 2.9. Evaluation of Physicochemical Properties of Q-SD-DMNs

#### 2.9.1. Penetration Efficiency

A penetration efficiency test was conducted to determine the ability of B-DMNs and Q-SD-DMNs to perforate a stack of Parafilm sheets, which represented skin barriers during insertion of microneedles for attaching a microneedle patch on the skin at an average pressing force [22,23]. 

In this study, eight sheets of Parafilm were vertically stacked and placed on the base of a texture analyzer (TA. XT Plus, Stable MicroSystems, Framingham, MA, USA) to obtain a total thickness of 1 mm, which is close to the thickness of the epidermis [24]. B-DMNs and the selected Q-SD-DMNs were attached to the compressing probe of the texture analyzer. Then, it was vertically lowered at a speed of 1 mm/s and applied a compression force of 32N to the stack of Parafilm sheets for 30 s. 

After being subjected to the test, the height of all B-DMNs and the selected Q-SD-DMN was observed under a light microscope (Nikon ECLIPSE Ts2R, Nikon Corporation, Tokyo, Japan) and compared to that of the intact needles before the test. A percentage of each needle height reduction was calculated by Equation (2) [10].

(2) $$\%\;\mathrm{Needle}\;\mathrm{height}\;\mathrm{reduction}=\frac{\mathrm{needle}\;\mathrm{height}\;(\mathrm{before})-\mathrm{needle}\;\mathrm{height}\;(\mathrm{after})\;}{\mathrm{needle}\;\mathrm{height}\;(\mathrm{before})}\times \;100$$

where the needle height (before) and needle height (after) are the height of each needle before and after being subjected to the test with the compression mode.

Thereafter, the number of needles that successfully penetrated through all the Parafilm sheets was calculated as an insertion percentage (% insertion) by counting the number of pores from needle perforation on the lowest Parafilm sheet using Equation (3).

(3) $$\;\%\;\mathrm{Insertion}=\frac{\mathrm{number}\;\mathrm{of}\;\mathrm{pores}\;\mathrm{on}\;\mathrm{the}\;\mathrm{lowest}\;\mathrm{Parafilm}\;\mathrm{sheet}}{\mathrm{number}\;\mathrm{of}\;\mathrm{total}\;\mathrm{arrays}}\times \;100$$

Since this experiment was a destructive test, three new microneedle patches of each formulation were investigated. The results are reported as a mean ± SD of these three samples.

#### 2.9.2. Determination of Quercetin Content in Q-SD-DMN

Quercetin content in the microneedle patch was determined from the needle part only. In this study, a film of the needle part of an optimized Q-SD-DMN as a representative was cast in a Petri dish with an accurate weight of Q-SD-DMN solution that was used for preparing complete needles with the same base dimension and all heights as previously reported after validations in triplicate. 

Thereafter, the film was dissolved in a mobile phase before being sonicated for 10 min, then centrifuged at 15,000 rpm for 10 min. The supernatant was filtered through a 0.45 µm membrane prior to the analysis by the HPLC technique.

#### 2.9.3. Evaluation of Physicochemical Properties of Q-SD-DMN

The physicochemical properties of the optimized Q-SD-DMN were evaluated using the PXRD, DSC, and FTIR techniques. Its morphology was also determined through SEM, as described in the previous sections.

#### 2.9.4. In Vitro Skin Permeation Study

The skin permeation study was performed to determine the permeability of quercetin from the optimized Q-SD-DMN and Q-DMN through neonatal porcine skins using a modified Franz diffusion cell [10]. Prior to the study, the needles of the optimized Q-SD-DMN and Q-DMN were analyzed for the quercetin content. Furthermore, the electrical resistance of the porcine skins was measured by using a multimeter (TMT460012 Total, Ping Shan, Hong Kong) to ensure its barrier function. The thickness of the porcine skin was also determined by using a Vernier caliper (IP6, Mitutoyo, Kanagawa, Japan). 

The microneedle patch was firmly pressed on the porcine skin, covering a diffusion area of 1.77 cm^2^, before it was placed into a receptor chamber. A receiving medium consisting of methanol (10% *v*/*v*), Tween 80 (0.4% *v*/*v*), and PBS (pH 7.4) was filled into the receptor chamber. It was continuously stirred using a magnetic stirrer at a temperature of 37 ± 1 °C. The receiving medium was withdrawn and replaced with fresh medium at the following times: 5, 15, 30 min, 1, 2, 3, 4, 5, 6, 7, and 8 h. 

The content of quercetin in the receiving medium was determined through HPLC analysis. The cumulative permeation of quercetin was plotted against time. Thereafter, a steady-state flux (J) was determined from a slope of the plots representing the amount of drug in the receptor chamber (q) at various times (t). The permeability coefficient (Kp) was then calculated using Equations (4) and (5) [25].

(4) $$\;J=\frac{\mathrm{dq}}{\;\mathrm{dt}\times A}$$

(5) $$\mathrm{Kp}=\frac{J}{\;\mathrm{Cd}}$$

where A and Cd represent the surface area of the mounted membrane and a concentration of quercetin in the microneedle patch, respectively.

The lag time of quercetin permeation was determined by extrapolating the steady-state portion of the graph to intersect the time axis. The study was conducted in three replications for each microneedle patch.

### 2.10. Investigation of Anti-Melanoma Activities of the Test Samples

#### 2.10.1. The Test Samples Preparation

The test samples, i.e., quercetin, the needle part of a representative Q-SD-DMN and B-DMN, were prepared using the same methods as reported in Section 2.9.2, were dissolved in the complete medium containing DMSO (0.25% *v*/*v*) to make different quercetin equivalent concentrations of 60, 80, 100, 150, 200, and 400 µg/mL. The untreated cells served as a control. 

#### 2.10.2. Cytotoxicity Test of the Test Samples by MTT Assay

The cytotoxicity of the test samples was determined using an MTT assay in the HFF-1 cells and A375 cells [26]. The cells were seeded in 96-well plates at a density of 0.01 × 10^6^ cells/well (100 μL/well) and incubated until reaching confluence. After that, they were treated with the test samples at various concentrations (100 μL/well) for 24 h. The cells were washed with PBS three times and incubated with 100 μL of MTT reagent (5 mg/mL) for 3 h. Subsequently, the reagent was removed, and the formazan crystals in the cells were solubilized with 100 μL of DMSO. The absorbance for each well was read at 570 nm using a microplate reader (Spectrostar Omega, BMG Labtech, Baden-Wurttemberg, Germany). Cell viability (CV) was calculated according to Equation (6):

(6) $$\mathrm{Cell}\;\mathrm{viability}\;(\%)=\frac{\mathrm{OD}\;\mathrm{sample}}{\mathrm{OD}\;\mathrm{control}}\times \;100$$

where OD sample and OD control are the OD of wells containing the cells incubated with and without the samples, respectively. The lowest concentration provided cell viability of more than 70%, and close to 100% was selected for further studies.

In addition, a dose-response graph was plotted between the concentrations of the test samples and the percentage of CV. The IC_50_ value, which indicates the concentration that inhibits 50% of CV, was determined. Finally, the selectivity index (SI) was calculated using Equation (7):

(7) $$\mathrm{SI}=\frac{\mathrm{IC}50\;\mathrm{of the samples in the HFF-1 cells}}{\mathrm{IC}50\;\mathrm{of}\;\mathrm{the}\;\mathrm{samples}\;\mathrm{in}\;\mathrm{the}\;A375\;\mathrm{cells}}$$

where the OD sample and OD control are the OD of wells containing the cells incubated with and without the test samples, respectively. 

#### 2.10.3. Apoptosis Analysis

The induction of apoptosis by the test samples in the A375 cells was investigated using a flow cytometry assay [27]. The A375 cells were seeded in 12-well plates at a density of 0.1 × 10^6^ cells/well (1000 μL/well) and incubated for 24 h. The medium was then replaced with fresh medium containing the test samples at the IC_50_ concentration and further incubated for 24 h at 37 °C in a 5% CO_2_ atmosphere. Subsequently, the cells were collected in a microfuge tube and resuspended in 1X binding buffer (500 μL). Annexin V-FITC (5 μL) and propidium iodide (PI) (5 μL) were added, followed by a 15 min incubation at room temperature in the dark. The samples were subsequently analyzed using a flow cytometry instrument (BD FACSVerse™, BD Biosciences, Milpitas, CA, USA) at an excitation/emission wavelength of 490/520 nm for Annexin V-FITC and 535/617 nm for PI.

#### 2.10.4. Evaluation of Bcl-2 Gene Expression Level in the A375 Cells

A reverse transcription-quantitative polymerase chain reaction (RT-qPCR) was employed to determine the effect of the test samples on the expression of the Bcl-2 gene in the A375 cells. The experiment was performed following the manufacturer’s instructions. Briefly, the cells were cultured in 6-well plates and exposed to the test samples at a selected concentration from the cell viability test for 24 h. Then, RNA was extracted from the cells and purified using the Bio-Rad Aurum Total RNA Mini Kit (Bio-Rad Laboratories Ltd., Hercules, CA, USA). The concentration of the RNA was determined using the LVis plate at a wavelength of 260/280 nm (a FLUOstar, BMG Labtech, Baden-Wurttemberg, Germany). The cDNA synthesis was performed using the iScript cDNA Synthesis Kit (Bio-Rad Laboratories Ltd., Hercules, CA, USA). The RT-PCR was conducted with 5 μL of iTaq Universal SYBR Green Supermix (Bio-Rad Laboratories Ltd., Hercules, CA, USA), 1 μL of cDNA, 1 μL of primer, and adjusted to a final volume of 10 μL with RNase/DNase-free water. 

The RT-qPCR was performed using primers of the target gene, Bcl-2, i.e., Prime PCR Assay, Bcl-2, (Cat. No. 10025636, Bio-Rad Laboratories, Hercules, CA, USA) and the housekeeping gene, i.e., 18S rRNA, (forward: GTAACCCGTTGAACCCCATT; reverse: CCATCCAATCGGTAGTAG CG). The reactions were conducted via a CFX96 Real-Time System with CFX Maestro V2 Software V.2.2. (Bio-Rad Laboratories Ltd., Hercules, CA, USA). The RT-qPCR running program included an initial enzyme activation step at 95 °C for 3 min, followed by 40 cycles of denaturation at 95 °C for 2 s and annealing at 60 °C for 20 s. The final extension phase consisted of 30 s at 50 °C, 5 s at 65 °C, and a final denaturation step at 95 °C for 5 s. 

All reactions were performed in triplicate. For relative expression analysis, the ΔΔCT method was used to compare gene expression levels between samples, typically employing a housekeeping gene for normalization. The calculations were performed as described by [28]. 

#### 2.10.5. Evaluation of Bcl-2 Expression Level in the A375 Cells

Western blot was applied to determine the expression of Bcl-2 in the A375 cells after they were exposed to the test samples. The A375 cells were cultured in 6-well plates and exposed to the test samples after they reached confluence for 24 h. Subsequently, the cells were washed with ice-cold PBS. A lysis buffer was then added to the wells. The A375 cells were incubated on ice for 5 min, followed by centrifugation of the mixture at 4 °C, 14,000 rpm for 15 min to collect the supernatant. The protein concentration was quantified using an LVis plate at a wavelength of 260/280 nm (FLUOstar plate reader, BMG Labtech, Baden-Wurttemberg, Germany). 

The samples containing 30 μg of total protein were mixed with sample loading buffer and then loaded into sodium dodecyl sulfate-polyacrylamide gel electrophoresis (SDS-PAGE). The gel was initially run at 50 V, 5 min, followed by 120 V, 75 min, using the PowerPac™ Basic Power Supply (Bio-Rad, USA). Thereafter, the proteins were transferred to a nitrocellulose membrane using a semi-dry transfer system (Trans-Blot Turbo Transfer System, Bio-Rad, USA). It was blocked with 5% non-fat dry milk in Tris-buffered saline with 0.05% Tween 20 for 1 h at room temperature. 

The membrane was then incubated with primary antibodies, anti-Bcl-2 (Cat. No. Ab59348, Abcam, Cambridge, UK) at a 1:1000 dilution, and anti-β-actin (Cat. No. Ab8227, Abcam, UK) at a 1:2500 dilution in a blocking buffer, 4 °C, 18 h. Afterward, it was incubated with horseradish peroxidase (HRP)-conjugated goat anti-rabbit IgG antibody (Cat. No. Ab6721, Abcam, UK) as a secondary antibody at a 1:3000 dilution for 1.5 h. The membrane was then exposed to an electrochemiluminescence (ECL) substrate and visualized using the ChemiDoc Imaging System (Bio-Rad, USA). Finally, the bands corresponding to Bcl-2 were analyzed using image J software V. 1.54f (NIH, Bethesda, MD, USA) to quantify protein expression in triplicate [29].

### 2.11. Statistical Analysis

The obtained data are presented as a mean ± standard deviation (SD). An independent *t*-test and a one-way ANOVA with Tukey’s post-hoc test were applied for statistical analysis at a significant level of 0.05 (SPSS Statistics software, V. 23, IBM, Chicago, IL, USA).

## 3. Results and Discussion

### 3.1. Preparation of Q-SDs

The percentage yields and content uniformity of Q-SDs prepared in this study are shown in Table 1. The results indicate a clear trend in the percentage yield of quercetin solid dispersions with varying ratios of quercetin to PVP. The yield of quercetin solid dispersions increased with the increase in the ratio of quercetin-to-PVP. It is worth noting that the percentage yields of all obtained Q-SDs were slightly low. This finding resulted from the loss of some water and some substances in the Q-SD mixtures during the freeze-drying process from sublimation [30]. However, the content uniformity of the obtained Q-SDs was still within a range of 90% to 110% of the theoretical amount (Table 1). Therefore, the preparation process of Q-SDs used in this study provided acceptable uniformity of Q-SDs [31]. The obtained Q-SDs were, thus, accepted for use in further studies.

### 3.2. Determination of Water Solubility of Quercetin

The results of the water solubility test of quercetin and quercetin in Q-SDs are shown in Table 1. It was found that the solubility of quercetin was significantly lower than that of quercetin in Q-SDs at the entire ratios of quercetin to PVP, i.e., 0.8:1, 1:1, 1.5:1, 1.8:1, and 2:1 at *p*-values of 0.000 for all ratios. This finding is due to the hydrophilic properties of PVP. It can form hydrogen bonds with the hydroxyl groups presenting in quercetin. They facilitated the uniform dispersion of quercetin molecules within the polymer matrix. This interaction prevents the arrangement of quercetin molecules from crystallization but maintains their amorphous form, which is more soluble in water when compared to their crystalline form [32]. It was found that the lower the ratios of quercetin to PVP, the higher the water solubility of quercetin. This finding is due to the fact that lower ratios result in a higher relative amount of PVP, which enhances the hydrogen bonding interactions between PVP and quercetin, thereby improving quercetin solubility [32].

Although Q0.8P1-SD exhibited the highest water solubility, it was not chosen for microneedle preparation. Since dissolving microneedles have limitations in drug loading capacity, the highest ratio of quercetin to PVP with significantly improved water solubility of quercetin when compared to that of quercetin, i.e., Q2P1-SD, was considered the most acceptable solid dispersion formulation for preparation of Q-SD-DMNs.

In this study, a physical mixture of quercetin and PVP at a ratio of quercetin-to-PVP = 2:1 (Q2P1-PM) was prepared. Its solubility in water was also determined and compared to the solubility of quercetin and quercetin in Q2P1-SD. It was found that the solubility of quercetin in Q2P1-PM was significantly higher than that of quercetin. However, it was lower than that of quercetin in Q2P1-SD at a *p*-value of 0.000 and 0.000, respectively. This result confirmed that the interaction between PVP and quercetin in the solid dispersion via hydrogen bonds facilitated the solubility of quercetin in water. Meanwhile, these interactions partially occurred in Q2P1-PM [32]. Therefore, the water solubility of quercetin in Q2P1-PM was slightly improved. This finding was consistent with the solubility enhancement of emamectin benzoate using the solid dispersion technique reported by Huang et al. [20]. 

#### 3.2.1. Results of the PXRD Analysis

**Figure 1:**
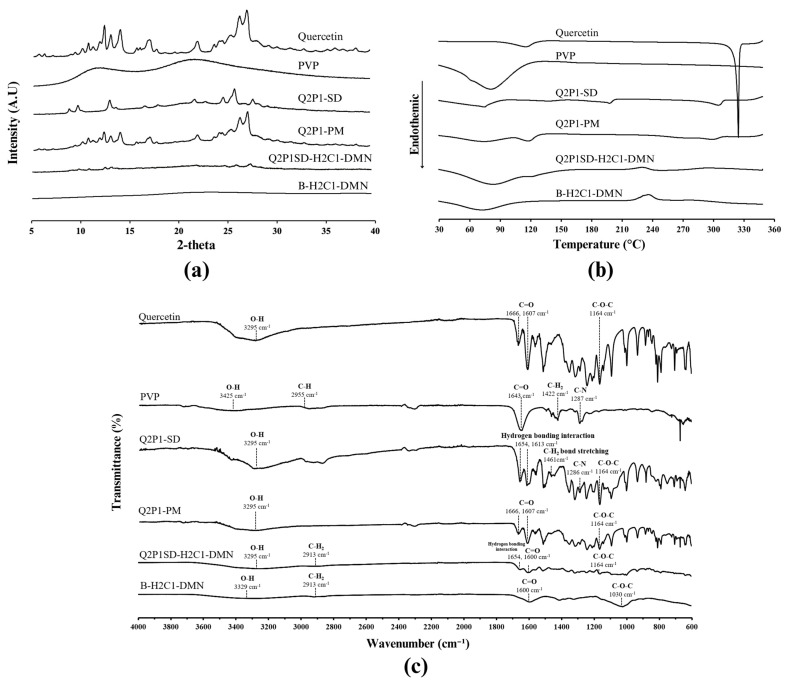
Characterization of quercetin, PVP, Q2P1-SD, Q2P1-PM, Q2P1SD-H2C1-DMN, and B-H2C1-DMN: (**a**) X-ray diffractograms, (**b**) DSC thermograms, (**c**) FTIR spectra.

The diffractogram of quercetin and PVP as raw materials, Q2P1-SD and Q2P1-PM, from the PXRD analysis are illustrated in Figure 1a. It was found that the diffractogram of quercetin shows diffraction peaks corresponding to the crystalline phase of quercetin at 5.76°, 6.24°, 9.48°, 10.24°, 10.78°, 11.34°, 12.42°, 13.10°, 14.08°, 15.84°, 17.20°, 17.92°, 22.16°, 26.56°, and 27.28° in 2θ. On the other hand, diffraction peaks are absent in the diffractogram of the PVP. This finding indicates an amorphous characteristic of PVP, which is consistent with the previous report by [33]. They found that all PVPs with various molecular weights (K15, K17, K30, and K60) did not show characteristic diffraction peaks within the scanning range. This finding confirmed the amorphous state of PVP.

When comparing the diffractograms of quercetin to that of quercetin in Q2P1-SD, some diffraction peaks were lost at some angles of diffraction, i.e., 5.76°, 6.24°, 10.24°, 10.78°, 11.34°, 13.10°, 14.08°, 17.20°, and 17.92°, along with trivial shifts of some diffraction peaks, i.e., 12.42°, 15.84°, 22.16°, 26.56°, and 27.28°. These findings pointed out a decrease in the crystalline characteristic of quercetin in Q2P1-SD.

However, most diffraction peaks in the diffractogram of quercetin were found in that of Q2P1-PM, which was a representative of physical mixtures and contained the same quercetin-to-PVP ratio as Q2P1-SD; they indicated a more crystalline characteristic when compared to that of Q2P1-SD. The lower crystallinity of quercetin in Q2P1-SD, thus, led to its significantly better water solubility when compared to that of Q2P1-PM and quercetin at *p*-values of 0.000 for both comparisons, as shown in the previous section. This finding was due to the interactions between the molecules of the crystalline quercetin and the amorphous PVP. They interrupted the recrystallization process of quercetin during freeze-drying and led to the absence of the three-dimensional molecular order of quercetin. These interactions also stabilized the metastable amorphous form of quercetin. Thus, they resulted in the amorphous Q2P1-SD [34]. 

The results from this study were consistent with a previous report by Wang et al. [21]. They also found that solid dispersion of quercetin in a PVP K30 matrix prepared by the solvent evaporation method at a ratio of quercetin-to-PVP K30 (1:3) exhibited amorphous characteristics of quercetin. 

#### 3.2.2. Results of the DSC Analysis

The thermogram of quercetin, PVP, Q2P1-SD, and Q2P1-PM from the DSC analysis is presented in Figure 1b. It shows a broad endothermic peak at 116 °C in a thermogram of quercetin, which indicated a thermal event, possibly related to either a phase transition or a loss of water [35]. This is due to the water molecules that are part of the crystal structure (crystal water) of the quercetin particles [36]. Furthermore, a sharp endothermic peak is observed at 326 °C, which is generally considered the melting point of quercetin. However, the previous study suggested that the DSC thermogram in the range of 150 to 350 °C of quercetin undergoes not only melt but also decomposition of quercetin’s structure. The authors indicated that the sharp peak at 326 °C represented both the melting process and the onset of thermal decomposition [35].

In contrast, sharp endothermic peaks could not be found in the thermogram of PVP; only a broad endothermic peak was shown at 81 °C. It indicated the loss of water from PVP, which is a hygroscopic amorphous polymer. This residual moisture caused a broad endothermic peak from 80 to 120 °C in the DSC curve [37]. 

From the thermogram of Q2P1-SD, broad endothermic peaks were found at 75 °C, 199 °C, and 307 °C. However, some endothermic peaks of those found in the thermogram of quercetin were absent in this thermogram. They implied that the crystallinity of quercetin in Q2P1-SD was suppressed by the interactions between the molecules of crystalline quercetin and the amorphous PVP during the solid dispersion process that led to molecular dispersion of quercetin in the PVP matrix [34]. The endothermic peak at 75 °C in the thermogram corresponded to the loss of water molecules from the hygroscopic PVP [38]. The broad peaks at 199 °C and 307 °C evidenced the formation of hydrogen bonds between quercetin and PVP during the thermal transformation and decomposition of quercetin molecules [21,35]. 

The thermogram of Q2P1-PM exhibited a broad endothermic peak at 116 °C. It indicated a thermal event related to the loss of water of crystallization in quercetin particles [35,36]. A broad endothermic peak around 300–307 °C, typically representing the melting point and thermal decomposition of quercetin, also pointed out the partial amorphous characteristics of quercetin in Q2P1-PM [35]. 

These results were consistent with the findings published by Setyawan et al. [39]. They found that both the physical mixture and solid dispersion of quercetin in hydroxypropyl methyl cellulose matrix exhibited a broad endothermic peak at 325.4 °C because of the loss of the crystalline characteristics of quercetin. They confirmed that quercetin in Q2P1-SD was in an amorphous form; meanwhile, quercetin in Q2P1-PM showed partial amorphous characteristics of quercetin. Consequently, the water solubility of quercetin in Q2P1-SD and Q2P1-PM was higher than that of quercetin. 

#### 3.2.3. Results of the FTIR Analysis

The FTIR spectra of quercetin, PVP, Q2P1-SD, and Q2P1-PM are presented in Figure 1c. The FTIR spectrum of quercetin exhibits characteristic peaks at wavenumbers of 1164, 1607 to 1666, and 3295 cm^−1^ corresponding to C−O−C vibration, C=O stretching, and O−H stretching, respectively [13,40]. Meanwhile, the FTIR spectrum of PVP shows identity peaks at a wavenumber of 1287, 1422, 1643, 2955, and 3425, which represent the C−N vibration, C−H_2_ stretching, C=O stretching, C−H stretching, and O−H stretching, respectively [40,41]. Regarding the FTIR spectrum of Q2P1-SD, some of the aforementioned peaks of quercetin are also presented at wavenumbers of 1164 and 3295 cm^−1^. However, the characteristic band of PVP with some trivial shifts was also found, for example, the band at wavenumbers of 1286, 1461, and 1654 cm^−1^. They evidenced the presentation of interactions between the molecules of quercetin and PVP in the solid dispersion matrix of Q2P1-SD, for example, the formation of hydrogen bonds between the hydroxyl groups of quercetin and the carbonyl groups of PVP [21].

The FTIR spectrum of Q2P1-PM shown in Figure 1c also indicates the presence of quercetin and PVP molecules. The characteristic bands of quercetin in the Q2P1-PM spectrum were observed at the same wavenumbers as those in the quercetin spectrum, without significant shifts, i.e., 1164 cm^−1^ (C−O−C stretching), 1607 cm^−1^ (C=O stretching), 1666 cm^−1^ (C=O stretching), and 3295 cm^−1^ (O−H stretching). This suggests that quercetin in Q2P1-PM was mainly in the form of physical dispersion, whereas quercetin in Q2P1-SD was molecularly dispersed in the PVP matrix.

### 3.3. Results of Morphology Observation

**Figure 2:**
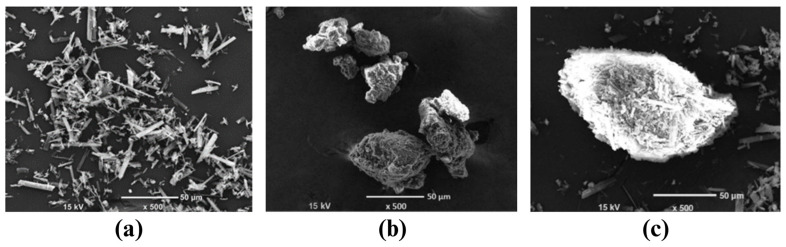
Morphology of (**a**) quercetin, (**b**) Q2P1-SD, and (**c**) Q2P1-PM.

The micrographs of quercetin powder, Q2P1-SD, and Q2P1-PM are illustrated in Figure 2. It shows a rod shape of quercetin crystals in Figure 2a. This finding indicated a crystalline property of quercetin powder. However, quercetin crystals could not be observed in Q2P1-SD, as shown in Figure 2b. This micrograph implied that the crystallinity of quercetin powder in Q2P1-SD was mostly absent. It was consistent with the results from the analysis of Q2P1-SD by the PXRD and DSC technique, as reported in Section 3.2.1 and Section 3.2.2. Figure 2b, thus, confirms that an amorphous form was a major form of quercetin in Q2P1-SD. 

On the other hand, crystals of quercetin powder were still found, as shown in Figure 2c. It is worth noting that Q2P1-PM mainly contained crystalline quercetin powder. This picture suggests that the physical dispersion of quercetin mainly occurred in Q2P1-PM. 

### 3.4. Development of Q-SD-DMN Formulations

**Figure 3:**
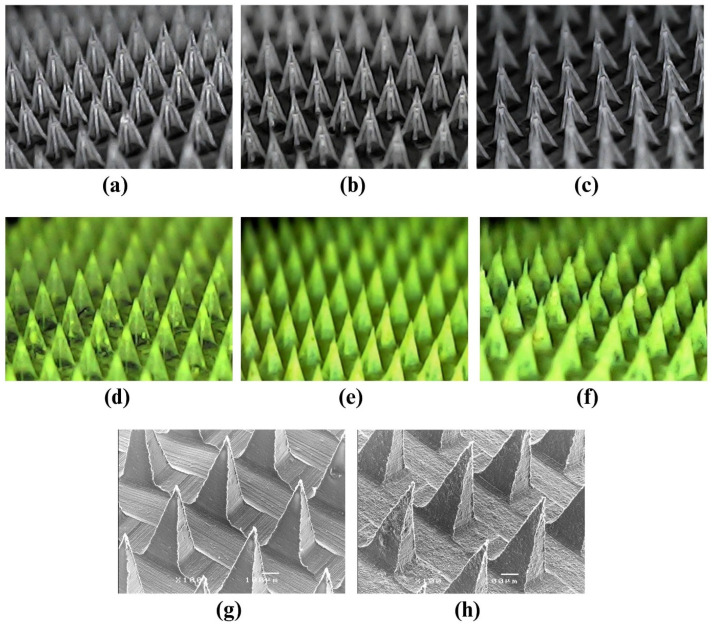
Morphology of microneedles: (**a**) B-H2C1-DMN, (**b**) B-H1C1-DMN, (**c**) B-H1C2-DMN; morphology of B-H2C1-DMN containing Q2P1-SD at various ratios of Q2P1-SD to the microneedle base, i.e., (**d**) 0.5:99.5, (**e**) 1:99, (**f**) 1.5:98.5; micrographs of (**g**) B-H2C1-DMN and (**h**) Q2P1SD-H2C1-DMN.

The formulations of Q-SD-DMNs developed in this study were based on the development of dissolving microneedle bases without the addition of quercetin (B-DMNs). They were prepared by varying the ratios of HA-to-Na-CMC, i.e., 2:1, 1:1, and 1:2, and cast in a mold to create 15 × 15 arrays of pyramid-shaped needles. The obtained products were B-H2C1-DMNs, B-H1C1-DMNs, and B-H1C2-DMNs regarding the ratio of HA-to-Na-CMC of 2:1, 1:1, and 1:2, respectively. The morphology of all B-DMNs shown in Figure 3a–c indicates that each microneedle patch is completely comprised of pyramid-shaped needles with smooth surfaces. Therefore, these obtained microneedle patches were subject to the penetration efficiency study.

#### 3.4.1. Penetration Efficiency Study of B-DMNs

**Figure 4:**
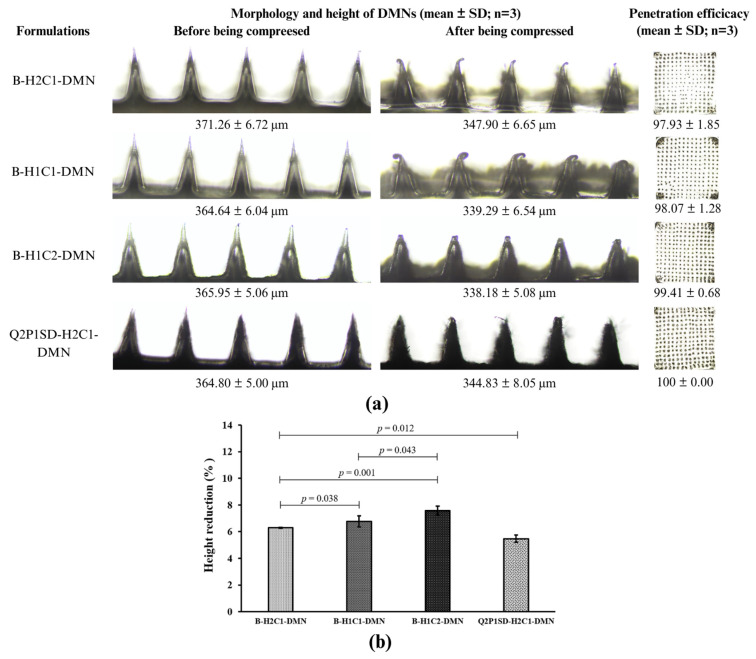
Characterization of DMNs under compression force (**a**) morphology and height of DMNs, (**b**) height reduction of B-DMNs and Q2P1SD-H2C1-DMNs.

The penetration efficiency study of the following microneedle patches, B-H2C1-DMN, B-H1C1-DMN, and B-H1C2-DMN, was conducted with a compression force of 32 N, which is an average compressing force for attaching the microneedle patch on the skin [23], by pressing the DMNs on a stack of Parafilm sheets. Thereafter, the height of the needles of each microneedle patch was measured. In Figure 4a, the mean of the initial heights of B-H2C1-DMN, B-H1C1-DMN, and B-H1C2-DMN were 371.26 ± 6.72, 364.64 ± 6.04, and 365.95 ± 5.06 µm, respectively. After the compression, the heights of the needles of the microneedle patches were reduced to 347.90 ± 6.65, 339.29 ± 6.54, and 338.18 ± 5.08, respectively. It was observed that the height reduction of the needles in all microneedle patches was mainly from distortion after being compressed, without the occurrence of fractures. This finding implied that the needles in the obtained microneedle patches were not brittle. They had enough strength to tolerate the compression force of 32 N with elasticity properties [24]. 

According to Figure 4b, the height reduction of B-H2C1-DMN exhibited the lowest value, which was statistically lower than that of B-H1C1-DMN and B-H1C2-DMN at a *p*-value of 0.038 and 0.001, respectively. These differences suggested that a higher ratio of HA-to-Na-CMC resulted in lower height reduction that represents the strength of the needles in the microneedle patches. This finding could be explained by the strong interactions between the polymer chains of HA and that of Na-CMC, for example, hydrogen bonding between the functional groups in HA and Na-CMC [42]. 

The penetration efficiency study of B-DMNs showed that B-H2C1-DMN, B-H1C1-DMN, and B-H1C2-DMN could perforate the lowest Parafilm sheet at the compression force of 32 N with all the needles contained in the microneedle patch. Their penetration efficiency was 97.93 ± 1.85%, 98.07 ± 1.28%, and 99.41 ± 0.68%, respectively, which was not significantly different at a *p*-value of 0.438. The results assured that these B-DMNs had enough strength to use for the development of Q-SD-DMNs.

#### 3.4.2. Preparation of Q-SD-DMNs

Q-SD-DMNs were prepared by gradually adding the optimized Q-SD, i.e., Q2P1-SD, into a solution of B-H2C1-DMN at three ratios of Q2P1-SD-to-B-H2C1-DMN as follows: 0.5:99.5, 1:99, and 1.5:98.5, respectively. The successful preparation of Q-SD-DMNs is demonstrated in Figure 3d–f. They revealed that Q-SD-DMNs at the ratios of 0.5:99.5 and 1:99 provided a complete microneedle patch with a regular pyramid-shaped structure of each needle. On the contrary, the formulation containing Q2P1-SD and B-H2C1-DMN at a ratio of 1.5:98.5 provided an irregular shape of the needles, as seen in the deformation of the needle tips in the microneedle patch. 

From these results, the formulation of Q2P1SD-H2C1-DMN containing Q2P1-SD and B-H2C1-DMN at a ratio of 1:99 was chosen as a representative for preparation of Q-SD-DMNs due to its high quercetin loading capacity. Furthermore, it could provide complete microneedles, as seen in Figure 3e. 

#### 3.4.3. Penetration Efficiency Study of Q-SD-DMNs

The penetration efficiency of Q2P1SD-H2C1-DMNs was also investigated with the same condition as the study of B-DMNs. Figure 4a illustrates the initial height of the Q2P1SD-H2C1-DMN. It was 364.80 ± 5.00 µm, meanwhile, the height after the compression test was 344.83 ± 8.05 µm. The statistical comparison shown in Figure 4b points out that the percentage height reduction of the Q2P1SD-H2C1-DMN was significantly less than that of B-H2C1-DMN, at a *p*-value of 0.012. Furthermore, the number of pores on the lowest Parafilm sheet from the compression of the Q2P1SD-H2C1-DMN was significantly more than that of the B-H2C1-DMN. This result suggested that the needles of Q2P1SD-H2C1-DMN were stronger and more flexible than those of B-H2C1-DMN because of the filling effect of Q2P1-SD leading to the higher interactions between the polymer chains and quercetin in Q2P1-SD in the microneedle [43,44]. Consequently, Q2P1SD-H2C1-DMN could resist the compression force more than B-H2C1-DMN [18].

#### 3.4.4. Characterization of Q2P1SD-H2C1-DMN

##### Results of the PXRD Analysis of Q2P1SD-H2C1-DMN

The PXRD diffractogram shown in Figure 1a illustrates the identity pattern of quercetin at the same angle of diffraction of quercetin in Q2P1-SD at 12.42°, 15.84°, 22.16°, 26.56°, and 27.28°, with lower intensity because of the small amount of quercetin in Q2P1SD-H2C1-DMN when compared to that of quercetin in Q2P1-SD. However, they were slightly different from those of the diffractogram of quercetin, i.e., 5.76°, 6.24°, 10.24°, 10.78°, 11.34°, 13.10°, 14.08°, 17.20°, and 17.92°. The shift of these angles of diffraction and the absence of some identity peaks that were found in the quercetin diffractogram indicated the incomplete crystalline characteristics of quercetin in Q2P1SD-H2C1-DMN. Meanwhile, none of the sharp diffraction peaks were found in the diffractogram of B-H2C1-DMN. This finding indicated an amorphous form of the polymer matrix consisting of B-H2C1-DMN. 

##### Results of the DSC Analysis of Q2P1SD-H2C1-DMN

The DSC thermogram of Q2P1SD-H2C1-DMN shown in Figure 1b shows the absence of the characteristic endothermic peak of quercetin at 326 °C that was found in the thermogram of quercetin and corresponded to its melting point [35]. This finding indicated the amorphous characteristic of quercetin in Q2P1SD-H2C1-DMN. Although the pattern of the thermogram of Q2P1SD-H2C1-DMN was similar to that of B-H2C1-DMN, because of the small content of quercetin in Q2P1SD-H2C1-DMN, only the broad identity peak of quercetin was found in the thermogram of Q2P1SD-H2C1-DMN at 126 °C, which corresponds to the transition temperature of quercetin [45]. It implied that quercetin consisting of Q2P1SD-H2C1-DMN was mainly in an amorphous form.

##### Results of the FTIR Analysis of Q2P1SD-H2C1-DMN

The FTIR spectrum of Q2P1SD-H2C1-DMN shown in Figure 1c indicates the presence of the characteristic bands of quercetin as seen in Q2P1-SD, at wave numbers of 1164, 1654, and 3295 cm^−1^, which represent C−O−C vibration, C=O stretching, and O−H stretching, respectively [13,40]. This finding indicated that quercetin had been presented in Q2P1-SD after incorporation into the B-H2C1-DMN matrix. 

It was found that some characteristic bands of HA and Na-CMC in B-H2C1-DMN, shown at wavenumbers 1030, 1600, 2913, and 3329 cm^−1^, corresponded to C−O−C symmetric stretching ether bands, C=O stretching, C−H stretching, and O−H stretching of both HA and Na-CMC [42], that could interact with the quercetin molecules via hydrogen bonds and Van der Waals interactions [46]. Some of these characteristic bands of HA and Na-CMC were also included in the spectrum of Q2P1SD-H2C1-DMN, i.e., 1600 and 2913 cm^−1^.

##### Results of the Morphology Observation of DMNs

Figure 3g,h illustrate the micrographs of B-H2C1-DMN and Q2P1SD-H2C1-DMN, respectively. They exhibit a sharp pyramidal shape of the needles in both microneedle patches. However, the needle surface of Q2P1SD-H2C1-DMN was not as smooth as that of B-H2C1-DMN. This finding resulted from the addition of Q2P1-SD into the matrix of the microneedle base. However, all needles in these obtained microneedle patches were complete without obvious flaws. The dimensions of needles in B-H2C1-DMN and Q2P1SD-H2C1-DMN, i.e., vertical height, slant height, and bases, were not significantly different at a *p*-value of 0.189, 0.099, and 0.528, respectively. This result suggested that the addition of Q2P1-SD did not affect the dimension of the needles in Q2P1SD-H2C1-DMN.

### 3.5. In Vitro Skin Permeation Study

Prior to the skin permeation test, the content of quercetin in the microneedles of the Q2P1SD-H2C1-DMN patch was quantified to ensure the consistency and effectiveness of the preparation process. The analysis revealed that the 225 needles (excluding the baseplate) contained 410.82 ± 1.99 µg of quercetin. 

**Figure 5:**
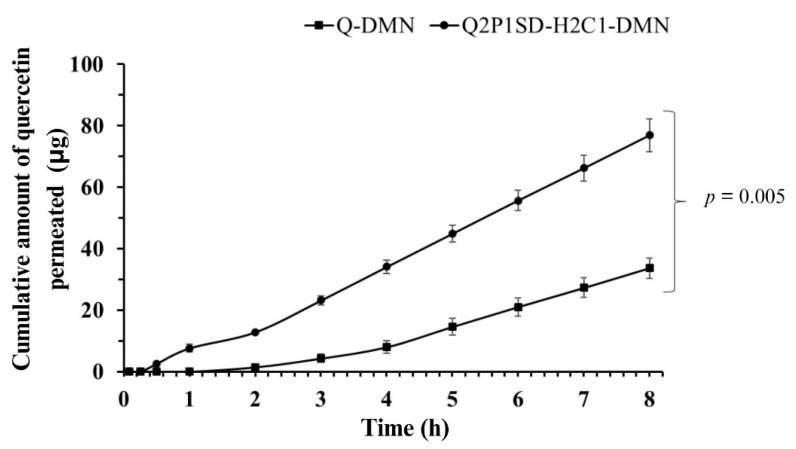
Cumulative permeation profiles of Q-DMN and Q2P1SD-H2C1-DMN in 8 h. Results are presented as a mean ± SD (n = 3). A significant difference is indicated at a *p*-value < 0.05.

The results of the in vitro skin permeation study are shown in Figure 5. It demonstrates that the skin permeation of quercetin content from Q2P1SD-H2C1-DMN was significantly higher than that of quercetin in Q-DMN, which was a DMN containing quercetin without being processed. The flux of quercetin from Q2P1SD-H2C1-DMN and Q-DMN were 4.76 ± 0.35 and 2.83 ± 0.31 μg/cm^2^/h, respectively. This finding could be explained by the higher solubility of quercetin in Q2P1SD-H2C1-DMN when compared to that of quercetin in Q-DMN. 

The results from this study also showed that quercetin from Q2P1SD-H2C1-DMN could permeate through the skin faster than that of quercetin from Q-DMN, which was represented as their lag time, i.e., 0.81 ± 0.04 h and 2.69 ± 0.42 h, respectively. This finding was also confirmed by the permeability coefficient (Kp) of quercetin from Q2P1SD-H2C1-DMN and Q-DMN, which was 0.012 ± 0.00 cm/h and 0.007 ± 0.00 cm/h, respectively. 

From these results, Q2P1SD-H2C1-DMN could be accepted as a promising delivery system for transdermal delivery of quercetin.

### 3.6. Anti-Melanoma Activities of Q-SD-DMNs

#### 3.6.1. Cytotoxicity Test

The cell viability (CV) of HFF-1 cells after treatment with quercetin, Q2P1SD-H2C1-DMN, and B-H2C1-DMN at various quercetin equivalent concentrations of 60, 80, 100, 150, 200, and 400 µg/mL were assessed using the MTT assay. 

**Figure 6:**
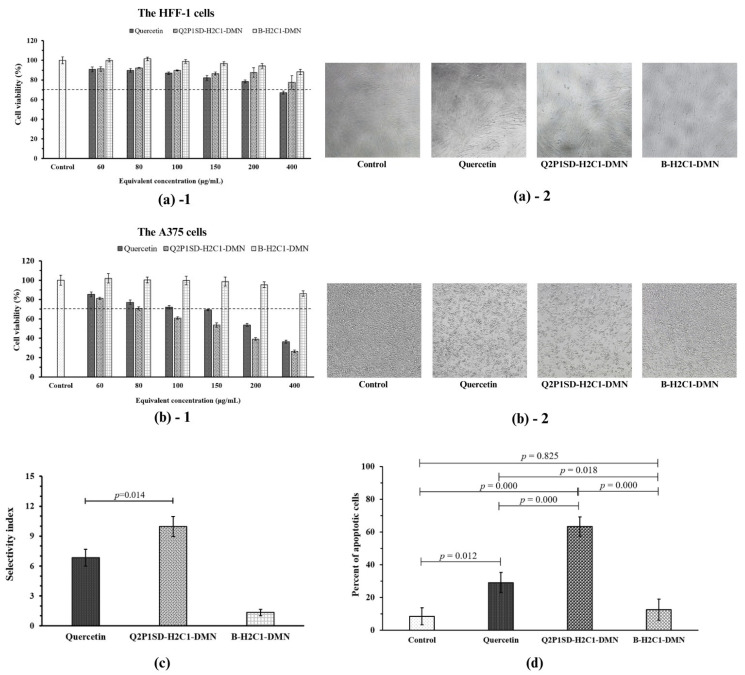
Bioactivities of quercetin, Q2P1SD-H2C1-DMN, and B-H2C1-DMN: (**a**)-**1** cell viability of the HFF-1 cells, (**a**)-**2** morphology of the HFF-1 cells after the incubation with the test samples at a concentration of 80 µg/mL, (**b**)-**1** cell viability of the A375 cells, (**b**)-**2** morphology of the A375 cells after the incubation with the test samples at a concentration of 80 µg/mL, (**c**) selectivity index of quercetin, Q2P1SD-H2C1-DMN, and B-H2C1-DMN, and (**d**) percentage of apoptotic cells. The results are presented as a mean ± SD (n = 3). The significant differences are indicated at a *p*-value < 0.05.

Figure 6a shows that quercetin exhibited a toxic effect on the HFF-1 cells with a CV lower than 70% at an equivalent concentration of 400 µg/mL. In contrast, Q2P1SD-H2C1-DMN at this concentration provided a CV of more than 70%. This lower cytotoxicity effect of Q2P1SD-H2C1-DMN was from the controlled release of quercetin from the HA and Na-CMC matrix in Q2P1SD-H2C1-DMN [47]. The gradual release of quercetin from the matrix maintained a slow rate of drug release over time. It, thus, prevented an immediate high concentration of the drug that could immediately lead to toxicity to the cells [48]. Additionally, the CV of the HFF-1 cells treated with B-H2C1-DMN was higher than 70% at entire equivalent concentrations (60–400 µg/mL), indicating that B-H2C1-DMN was not toxic to the HFF-1 cells at these concentrations.

Figure 6b illustrates the CV of the A375 cells treated with quercetin, Q2P1SD-H2C1-DMN, and B-H2C1-DMN at the same equivalent concentrations as the concentrations for the HFF-1 cells. The MTT assay was then performed. It was found that quercetin exhibited significant cytotoxicity to the A375 cells at concentrations of 150 to 400 µg/mL. This finding is consistent with the known anti-proliferative and pro-apoptotic effects of quercetin on melanoma cells [49]. Q2P1SD-H2C1-DMN exhibited a more gradual reduction in CV when compared to that of quercetin, with a CV of 70% at an equivalent concentration of 80 µg/mL. When the concentrations were increased, a decrease in the CV of the cells was observed. The CV of the A375 cells was lower than 70% at the equivalent concentration of equal and more than 100 µg/mL. B-H2C1-DMN demonstrated more than 70% of CV at all equivalent concentrations (60–400 µg/mL). This finding implied that the entire equivalent concentrations of B-H2C1-DMN were not toxic to the A375 cells.

It is worth noting that Q2P1SD-H2C1-DMN provided a lower CV of the A375 cells than that of quercetin because of the synergistic effect of HA-based dissolving microneedles. HA could facilitate the cytotoxicity of quercetin through an increase in cellular uptake by targeting CD44 receptors [50,51]. When HA binds to the CD44 receptors on the surface of melanoma cells, it promotes a greater influx of quercetin into cancer cells, effectively inhibiting cell proliferation and inducing apoptosis [51].

Since 80 µg/mL was the concentration at which all test samples provided 70% CV in A375 cells, this concentration was used for further experiments.

Figure 6c presents the selectivity index (SI) of quercetin, Q2P1SD-H2C1-DMN, and B-H2C1-DMN, i.e., 6.83 ± 0.83, 9.97 ± 1.01, and 1.33 ± 0.31, respectively. It was found that Q2P1SD-H2C1-DMN had significantly higher SI than that of quercetin (a *p*-value = 0.014). It implied that Q2P1SD-H2C1-DMN had promising inhibitory activities against the A375 cells; meanwhile, it was not toxic to the HFF-1 cells. These findings resulted from the activities of quercetin in Q2P1SD-H2C1-DMN, which can protect the cells from oxidative stress in the normal cells and induce apoptosis in the cancer cells [16]. However, the SI of quercetin and B-H2C1-DMN were lower than that of Q2P1SD-H2C1-DMN because the cells exposed to the high concentration of quercetin directly without controlled release quercetin from the microneedles, thus, it caused a hazardous environment toxic to the cells. The low SI value of B-H2C1-DMN was due to B-H2C1-DMN not containing active compounds to suppress the cancer cells. Therefore, B-H2C1-DMN was not toxic to both the HFF-1 and the A375 cells.

#### 3.6.2. Results of the Apoptosis Analysis

Figure 6d illustrates the percentage of apoptotic cells of the A375 cells treated with quercetin, Q2P1SD-H2C1-DMN, and B-H2C1-DMN at a concentration of 80 µg/mL, which resulted in 70% CV. Quercetin significantly increased the percentage of apoptotic cells in the A375 cells when compared to that of the A375 cells in the control (*p*-value = 0.012) and that of the cells treated with B-H2C1-DMN (*p*-value of 0.018). 

However, it induced fewer apoptotic cells compared to that of Q2P1SD-H2C1-DMN (*p*-value = 0.000). This finding could be explained by the fact that the controlled release of quercetin from the microneedle containing HA and Na-CMC improved cellular uptake of quercetin into the cells through the enhancement of HA molecules targeting the CD44 receptors in the A375 cells [47,50,51]. 

Furthermore, Q2P1SD-H2C1-DMN induced a higher percentage of apoptotic cells when compared to that of the cells induced by B-H2C1-DMN (*p*-value = 0.000). This result was from the absence of quercetin in B-H2C1-DMN. Therefore, the percentage of apoptotic cells induced by B-H2C1-DMN was comparable to that of the cells in the control (*p*-value = 0.825). 

#### 3.6.3. Results of the RT-qPCR Analysis

An RT-qPCR analysis was performed to investigate the molecular effect of quercetin in Q2P1SD-H2C1-DMN on Bcl-2 gene expression in the A375 cells, which is a gene responsible for apoptosis induction in the cancer cells. In this study, the A375 cells were treated with quercetin, Q2P1SD-H2C1-DMN, and B-H2C1-DMN at the equivalent concentration of 80 µg/mL.

**Figure 7:**
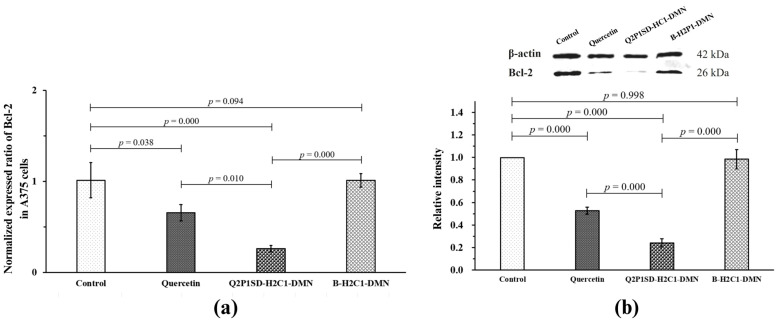
Effect of quercetin, Q2P1SD-H2C1-DMN, and B-H2C1-DMN on Bcl-2 gene expression in the A375 cells: (**a**) normalized expression ratios of Bcl-2 to 18S rRNA; and (**b**) relative intensity of Bcl-2 expression (mean ± SD, n = 3).

From Figure 7a, it is found that the A375 cells treated with quercetin and Q2P1SD-H2C1-DMN showed a significant decrease in Bcl-2 gene expression, with *p*-values of 0.038 and 0.000, respectively, when compared to that of the control. This result indicated that both quercetin and Q2P1SD-H2C1-DMN exhibited downregulation activities on Bcl-2 gene expression. However, Q2P1SD-H2C1-DMN could more suppress the expression of the Bcl-2 gene than quercetin (*p*-value = 0.010). This effect was due to the controlled release of quercetin from Q2P1SD-H2C1-DMN and more effective cellular uptake enhancement via targeting CD44 receptors by HA in the microneedle matrix [47,50,51]. Conversely, the Bcl-2 gene expression in the A375 cells treated with B-H2C1-DMN was statistically comparable with the cells in the control (*p*-value = 0.094). This finding evidenced that the suppressive activity of Q2P1SD-H2C1-DMN on Bcl-2 gene expression was from quercetin in the microneedle, leading to the downregulation of Bcl-2 gene levels in the A375 cells [52].

#### 3.6.4. Results of the Western Blot Assay

The effects of quercetin, Q2P1SD-H2C1-DMN, and B-H2C1-DMN on the expression of the Bcl-2 gene were confirmed by the Western blot analysis to determine Bcl-2 protein expression levels after treatment of the A375 cells with the test samples. The relative intensity of the Bcl-2 protein was quantified and normalized to the level of β-actin protein. As illustrated in Figure 7b, the control A375 cells without any treatments exhibited the highest levels of Bcl-2 protein. 

However, the treatment with quercetin and Q2P1SD-H2C1-DMN significantly reduced the Bcl-2 levels when compared to that of the control at a *p*-value of 0.000 and 0.000, respectively. The results indicated that both quercetin and Q2P1SD-H2C1-DMN could promote the cell death of the A375 cells via suppression of Bcl-2 gene expression [52]. Meanwhile, B-H2C1-DMN did not show suppressive activities against the Bcl-2 protein level. This finding was due to the fact that B-H2C1-DMN did not possess inhibitory activities on the Bcl-2 gene expression, leading to the comparable Bcl-2 protein level to that of the control.

It is worth noting that Q2P1SD-H2C1-DMN showed stronger suppressive activities on Bcl-2 protein expression than that of quercetin because of the optimized solubility of quercetin in the microneedles that were prepared in the form of solid dispersion. Furthermore, the polymer matrix of the microneedles not only provided a gradual release of quercetin from Q2P1SD-H2C1-DMN but also facilitated cellular uptake via the action of HA, as discussed in the previous section.

## 4. Conclusions

Formulations of SD-DMNs were successfully developed in this study. They contained Q-SD that was prepared by a solvent technique before being incorporated into the microneedle bases. The optimized formulation of SD-DMN was Q2P1SD-H2C1-DMN containing Q-SD with a ratio of 2:1 quercetin-to-PVP. It also consisted of HA and Na-CMC at a ratio of 2:1 as a microneedle base. The PXRD and DSC analysis indicated that quercetin in Q-SD and Q2P1SD-H2C1-DMN were mainly in an amorphous form. This characteristic of quercetin thus promoted its water solubility. The results from FTIR analysis pointed out the interactions between quercetin and the ingredients in either Q-SD or SD-DMN; therefore, the crystallinity of quercetin in both Q-SD and SD-DMN was decreased. The in vitro skin permeation test revealed that Q2P1SD-H2C1-DMN promoted the flux of quercetin with a higher skin permeation rate than those of Q-DMN. It was found that Q2P1SD-H2C1-DMN had inhibitory activities on the A375 cells via the induction of apoptosis in these melanoma cells. Importantly, Q2P1SD-H2C1-DMN effectively suppressed the activity of Bcl-2 by downregulation of Bcl-2 gene expression and led to low levels of Bcl-2 in the cells.

Therefore, Q2P1SD-H2C1-DMN has the potential for application as a transdermal delivery system of quercetin to induce apoptosis in melanoma cells. In addition, it can be accepted for further in vivo study in animal models to determine its safety and efficacy as a synergistic treatment for melanoma. 

## Figures and Tables

**Table 1:** Yield, content uniformity, water solubility of Q-SDs (mean ± SD; n = 3).

Q-SDs	Quercetin/PVP Ratios	Yield (%)	Content Uniformity (%)	Water Solubility (μg/mL)
Quercetin *	1:0	ND **	ND **	2.61 ± 0.01
Q0.8P1-SD	0.8:1	4.30 ± 0.15	96.90 ± 0.77	72.55 ± 0.34
Q1P1-SD	1:1	4.69 ± 0.14	96.48 ± 1.84	71.21 ± 0.29
Q1.5P1-SD	1.5:1	5.87 ± 0.05	97.82 ± 1.03	54.38 ± 0.08
Q1.8P1-SD	1.8:1	6.58 ± 0.13	97.26 ± 2.27	25.87 ± 0.08
Q2P1-SD	2:1	6.94 ± 0.03	97.76 ± 0.67	22.05 ± 0.08
Q2P1-PM	2:1	ND	98.24 ± 1.07	12.59 ± 0.15

* Quercetin is a raw material used for Q-SD preparation. ** ND = not determined.

## Data Availability

Data is unavailable due to privacy restriction.

## Abbreviations

B-Ds	Base formulation of dissolving microneedles
DEGEE	Diethylene glycol monoethyl ether
HA	Hyaluronic acid
Na-CMC	Sodium carboxymethylcellulose
PBS	Phosphate buffered saline
PVP	Polyvinylpyrrolidone K30
Q-DMNs	Quercetin-loaded dissolving microneedles
Q-PM	Quercetin physical mixture
Q-SDs	Quercetin solid dispersions
Q-SD-DMNs	Quercetin solid dispersion-loaded dissolving microneedles
SD-DMNs	Solid dispersion-loaded dissolving microneedles
SSM	Superficial spreading melanoma
